# The biodistribution and clearance of AlbudAb, a novel biopharmaceutical medicine platform, assessed via PET imaging in humans

**DOI:** 10.1186/s13550-019-0514-9

**Published:** 2019-05-21

**Authors:** Kevin S. Thorneloe, Armin Sepp, Sean Zhang, Laura Galinanes-Garcia, Paul Galette, Wasfi Al-Azzam, Danielle J. Vugts, Guus van Dongen, Phillip Elsinga, Johan Wiegers, Andor W. J. M. Glaudemans, Veena Vincent, Jessica Renaux, Matt Szapacs, Mary Birchler, Matthew Cleveland, Mats Bergstrom, Marie Davies

**Affiliations:** 1 GSK, South Collegeville Road, Collegeville, PA 19426 USA; 2 GSK, Gunnels Wood Road, Stevenage, Herts SG1 2NY UK; 3 VU University Medical Center, 1081 HV Amsterdam, The Netherlands; 4 University of Groningen, University Medical Center Groningen, Department of Nuclear Medicine and Molecular Imaging, Groningen, The Netherlands

**Keywords:** Domain antibody, Albumin, AlbudAb, PET/CT, Biodistribution, ^89^Zr

## Abstract

**Abstract:**

Conjugation or fusion to AlbudAbs™ (albumin-binding domain antibodies) is a novel approach to extend the half-life and alter the tissue distribution of biological and small molecule therapeutics. To understand extravasation kinetics and extravascular organ concentrations of AlbudAbs in humans, we studied tissue distribution and elimination of a non-conjugated ^89^Zr-labeled AlbudAb in healthy volunteers using positron emission tomography/computed tomography (PET/CT).

**Methods:**

A non-conjugated AlbudAb (GSK3128349) was radiolabeled with ^89^Zr and a single 1 mg (~ 15 MBq) dose intravenously administered to eight healthy males. ^89^Zr-AlbudAb tissue distribution was followed for up to 7 days with four whole-body PET/CT scans. ^89^Zr-AlbudAb tissue concentrations were quantified in organs of therapeutic significance, measuring standardized uptake value and tissue/plasma ratios. Plasma pharmacokinetics were assessed by gamma counting and LC-MS/MS of blood samples.

**Results:**

^89^Zr-AlbudAb administration and PET/CT procedures were well tolerated, with no drug-related immunogenicity or adverse events. ^89^Zr-AlbudAb rapidly distributed throughout the vasculature, with tissue/plasma ratios in the liver, lungs, and heart relatively stable over 7 days post-dose, ranging between 0.1 and 0.5. The brain tissue/plasma ratio of 0.025 suggested minimal AlbudAb blood-brain barrier penetration. Slowly increasing ratios in muscle, testis, pancreas, and spleen reflected either slow AlbudAb penetration and/or ^89^Zr residualization in these organs. Across all tissues evaluated, the kidney tissue/plasma ratio was highest (0.5–1.5 range) with highest concentration in the renal cortex. The terminal half-life of the ^89^Zr-AlbudAb was 18 days.

**Conclusion:**

Evaluating the biodistribution of ^89^Zr-AlbudAb in healthy volunteers using a low radioactivity dose was successful (total subject exposure ~ 10 mSv). Results indicated rapid formation of reversible, but stable, complexes between AlbudAb and albumin upon dosing. ^89^Zr-AlbudAb demonstrated albumin-like pharmacokinetics, including limited renal elimination. This novel organ-specific distribution data for AlbudAbs in humans will facilitate a better selection of drug targets to prosecute using the AlbudAb platform and significantly contribute to modeling work optimizing dosing of therapeutic AlbudAbs in the clinic.

**Electronic supplementary material:**

The online version of this article (10.1186/s13550-019-0514-9) contains supplementary material, which is available to authorized users.

## Introduction

Fully human heavy and light chain variable domain antibodies (dAbs) display good biochemical, biophysical, and antigen-binding affinities [1–3], but their low molecular weight (10–15 kDa) renders them susceptible to rapid renal clearance that limits their therapeutic potential. While this aspect has been overcome with albumin-binding dAbs, as exemplified by Exendin-4 AlbudAb demonstrating a reduction in glucose and insulin in humans, very little is known about the tissue penetration properties of AlbudAbs™ in humans. This information is critical to modeling tissue concentrations, target engagement, and thereby making predications for clinical efficacy.

GSK3128349, a member of the AlbudAb platform, is a light chain dAb with high affinity for human albumin (Kd = 0.85 nM) and can be conjugated or fused to a therapeutic biologic or small molecule to extend their plasma half-lives. Albumin is found at high concentrations in plasma, but also in extravascular spaces [4, 5], and therefore AlbudAb tissue distribution is likely to extend beyond the blood. In combination with plasma concentration measurements, positron emission tomography/computed tomography (PET/CT) [6–8] provides the capability to explore not only the steady-state, but importantly also the kinetics of AlbudAb extravasation. Understanding the kinetics provides the opportunity to assess the turnover of AlbudAb in extravascular organ compartments, and estimate the level of target engagement with respect to dose and time [9].

Positron emission tomography (PET) imaging of proteins requires measurement of a slower tissue penetration rate and longer plasma exposure than typical for small molecule drugs. Proteins do not diffuse across plasma membranes and extravasation predominantly takes place via paracellular pores. Zirconium-89 (^89^Zr), with a half-life of 3.3 days, is therefore a radiolabel of choice for protein PET studies, remaining detectable for up to 10 days even at the low dose levels which are deemed appropriate in clinical studies [10, 11]. Protein labelling methods for ^89^Zr utilize conjugation of desferrioxamine derivates and form very stable Zr complexes [10, 12, 13] .

In the present study, we administered ^89^Zr-GSK3128349 AlbudAb to eight healthy male volunteers and used a combination of PET/CT, scintillation counting, and mass spectrometry in an adaptive design to follow the time course of AlbudAb tissue and plasma concentrations simultaneously. These results will facilitate quantitative insight into target accessibility for AlbudAbs across various human organs and improve the likelihood of success in developing AlbudAb-based therapeutics.

## Materials and methods

### Subjects

Eight healthy male participants between 50 and 65 years of age were enrolled. The higher age range was motivated to limit lifetime cancer risk from radiation exposure, as outlined by the International Commission on Radiological Protection [11]. The body mass index (BMI) for the participants ranged between 19.0 and 31.0 kg/m^2^, with full details given in the Additional file 1. The study was approved by the appropriate regulatory and ethics committees (UMCG Medisch Ethische Commissie, Groningen, Netherlands) as required, and conducted in accordance with the International Conference on Harmonization for Good Clinical Practice and the principles of the Declaration of Helsinki. It was monitored by an Internal Safety Review Committee. Informed consent was obtained from all individual participants included in the study. The study was listed on ClinicalTrials.gov (NCT02829307).

### AlbudAb production and radiolabeling

GSK3128349 was manufactured and tested to ensure quality, identity, potency, and safety in accordance with current GSK Good Manufacturing Practices (cGMP). ^89^Zr was coupled to unlabeled GSK3128349 via the bifunctional chelate desferal at the VU University Medical Center [13]. See Additional file 1. Procedures for radiolabelling of GSK3128349 with ^89^Zr were validated with respect to stepwise quality controls and assessment of final product quality. A single batch of GSK3128349 was used for production of all ^89^Zr-GSK3128349 radiolabeled batches.

^89^Zr-GSK3128349 was diluted with unlabeled GSK3128349 to achieve a specific activity of 15 MBq/mg, with the ^89^Zr-GSK3128349 constituting around 0.015% of the total 1 mg GSK3128349 protein administered. A 1 mg total dose was utilized, as based on the pharmacokinetic properties of GSK3128349 it was deemed the minimum dose required to enable measurement of GSK3128349 plasma concentrations by mass spectrometry.

### Study design

Participants were screened up to 28 days before entering the clinic, when on day 1 a single intravenous (IV) dose of 1 mg of 15 MBq/mg ^89^Zr-GSK3128349 was administered (Additional file 1: Figure S1). Participants were discharged from the clinic on day 2, and overall underwent four PET/CT scans within the 7-day post-dose period. Multiple blood samples were taken for up to 42 days post-dosing to analyze plasma radioactivity, GSK3128349 concentration, and presence of anti-GSK3128349 antibodies.

Using data from the first two individuals, the organ and whole-body radiation exposure was calculated using OLINDA software to confirm that the effective doses were below 10 mSv (as outlined by the International Commission on Radiological Protection) [11]. As a result, no reduction in administered radioactivity was deemed necessary for the remaining six participants. Anonymised individual participant data and study documents can be requested for further research from www.clinicalstudydatarequest.com.

### PET procedures and analysis

The exact amount of administered radioactivity was determined by measurements of the injection syringe before and after administration. The IV infusion of 10 mL was administered over approximately 20 min. All scanning and blood sampling times refer to start of the infusion.

The PET scanning performed from head to mid-thigh was done using two EARL accredited Biograph mCT 64 slice PET/CT systems from Siemens, Knoxville, USA. The scanning time per bed position was typically 5 min but increased to 9 min in the later scans to enhance detection. Images were reconstructed with full corrections for attenuation and scatter, including a post-processing filter of 6.5 mm giving a resolution of 7 mm. The reconstructions were done including PSF (point-spread function) and TOF (time-of-flight) and conform to the current EARL specifications. The low-dose CT was performed at each PET scanning session for attenuation correction with settings to allow for no more than 0.5 mSv per session.

In the PET images, volumes of interest (VoI) were outlined in the major organs, using an ellipsoid fitting inside the organ, avoiding border effects. The average radioactivity concentration was determined and represented as SUVmean (SUV corrected for injected radioactivity and body weight). For smaller organs or structures, the principle of SUVpeak was applied, averaging over a sphere of 1 cm^3^. For some organs with uncertain visibility in the PET images, the delineation was aided by the CT scan. All SUV values were decay corrected to the start time of tracer administration.

The lowest theoretical value for the tissue-plasma ratio ${\left(\frac{t}{p}\right)}_{\mathrm{min}}$ is calculated from Eq. 1,

1 $$ {\left(\frac{t}{p}\right)}_{\mathrm{min}}=\frac{V_{P, org}}{V_{org}} $$

, where *V*_*P*, *org*_ is the organ plasma volume and *V*_*org*_ the total volume. The highest theoretical value for the tissue-plasma ratio ${\left(\frac{t}{p}\right)}_{\mathrm{max}}$ corresponds to the situation where the interstitial concentration is equal to that in plasma and calculated from Eq. 2,

2 $$ {\left(\frac{t}{p}\right)}_{\mathrm{max}}=\frac{V_{P, org}+{V}_{I, org}}{V_{org}} $$

, where *V*_*I*, *org*_ is the organ interstitial volume. All respective volume values were taken from the parameter set compiled by Shah and Betts [14].

### Pharmacokinetics

Plasma samples were collected for the first two subjects and encompassed pre-dose, 1, 3, 6, 8 h and 1, 2, 3, 5, and 12 days after administration, respectively, and analyzed using gamma counting and liquid chromatography tandem mass spectrometry (LC-MS/MS) for quantitation of GSK3128349. Additional samples were taken at 19, 30, and 43 days, but only analyzed with respect to total GSK3128349 using LC-MS/MS. The timing of the blood sampling for the remaining subjects was modified as determined most appropriate from observation of data from the first two subjects. Blood samples were always taken in conjunction with PET scanning. ^89^Zr-GSK3128349 plasma radioactivity was measured in a well counter cross-calibrated with the camera and decay corrected to the start of administration. Plasma concentrations of total GSK3128349 were determined by trypsin digestion and solid-phase extraction of the specific LLILAFSR peptide fragment derived from the AlbudAb complementarity determining region, which was quantified by LC-MS/MS analysis [15].

Cumulative urine was collected for the first 24 h post-dosing to determine the percent of administered radioactivity recovered in urine. If the total amount of radioactivity excreted in urine exceeded 3% for the first two subjects, then further discrete urine samples would be taken for subsequent subjects.

Non-compartmental analysis (NCA) of the plasma pharmacokinetics of both ^89^Zr-labeled as well as unlabeled GSK3128349 AlbudAb was performed using Matlab 2017b SimBiology v5.7.

### Safety and immunogenicity

Physical examinations, medical history, ECG, and clinical laboratory measurements were performed before and after dosing GSK3128349 on day 1 and on day 2. Routine hematology, serum chemistry, and urine analysis were performed on day − 1, 2, 6, 13 (± 1), 20 (± 2), and 21 (± 2). The presence of antibodies to GSK3128349 was assessed using an analytically validated bridging electrochemiluminescent (ECL) immunoassay. Briefly, serum samples collected on day 1 (before dosing) or day 43 (± 2; post dosing) were incubated for 1 h with biotinylated GSK3128349 and sulfo-TAG-labeled GSK3128349 before addition to a streptavidin-coated MSD plate. After 1 h, the plate was washed (PBS-tween) and read on a MSD Sector Imager Reader 600. Normal human serum and serum spiked with an anti-IgG (Vk) mAb were included as negative and positive controls, respectively.

## Results

### Subjects and safety

Eight subjects were enrolled into the study (Table 1), with inclusion/exclusion criteria listed in the Additional file 1. The mean administered radioactivity was 14.0 ± 0.9 MBq (range, 12.77–15.03 MBq). The effective dose from administered radioactivity for the first two subjects were 5.4 and 7.5 mSv, which together with four low dose CT scans (0.5 mSv/scan) gave total effective radiation doses of 7.4 and 9.5 mSv. The average radiation exposure across all study subjects was 0.448 mSv/MBq, with a mean subject exposure of 8.3 mSv.

**Table 1:** Eight Caucasian male subjects were enrolled into the study. All subjects had four PET/CT scans, except subject 122 who declined further participation after the first two scanning sessions for availability reasons

Subject	Age (years)	Weight (kg)	BMI (kg/m^2^)	Dose of radioactivity (MBq)
105	54	76.1	25.7	12.77
110	53	72.2	22.8	14.53
122	54	68.6	23.5	14.24
123	60	92.3	27.6	13.27
125	53	91.8	27.4	14.64
127	56	70.2	21.4	14.72
132	54	73.0	20.9	15.03
135	56	83.1	23.3	13.06

^89^Zr-GSK3128349 was well tolerated and no adverse events related to the AlbudAb were observed in any of the subjects. No observed changes in vital signs, laboratory results, or electrocardiograms were deemed clinically significant. One subject was found to have pre-existing anti-GSK3128349 antibodies at the start of study. In this subject, the titres were low and similar at follow-up, suggesting that these anti-GSK3128349 antibodies were not related to dosing. None of the subjects developed anti-GSK3128349 responses during the study.

### PET/CT images

**Fig. 1:**
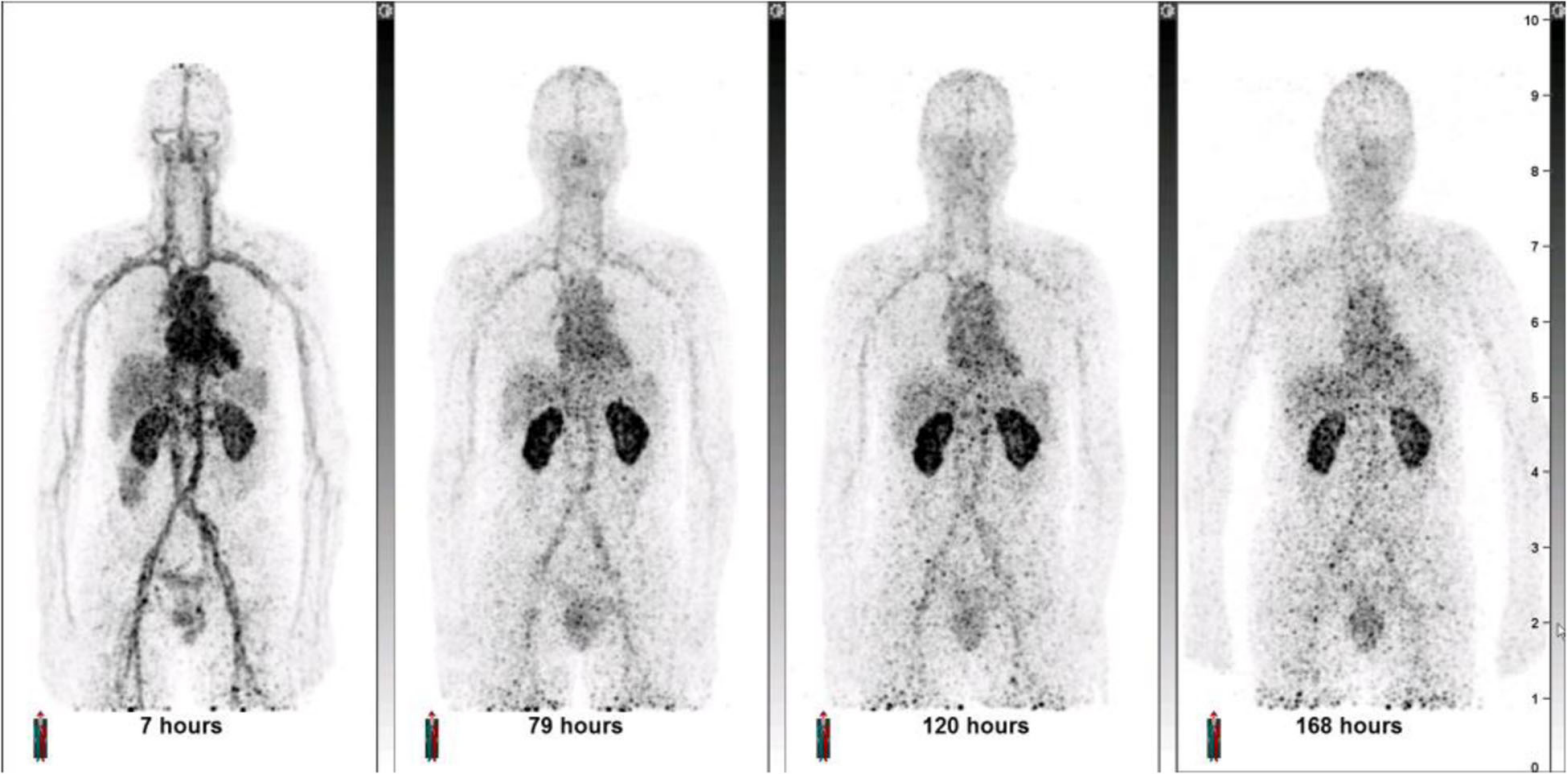
Maximum intensity projections (MIP) obtained at different time points after administration for one representative subject 135. MIP images for additional subjects 105 and 127 are provided in the Additional file 1: Figures S2 and S4

**Fig. 2:**
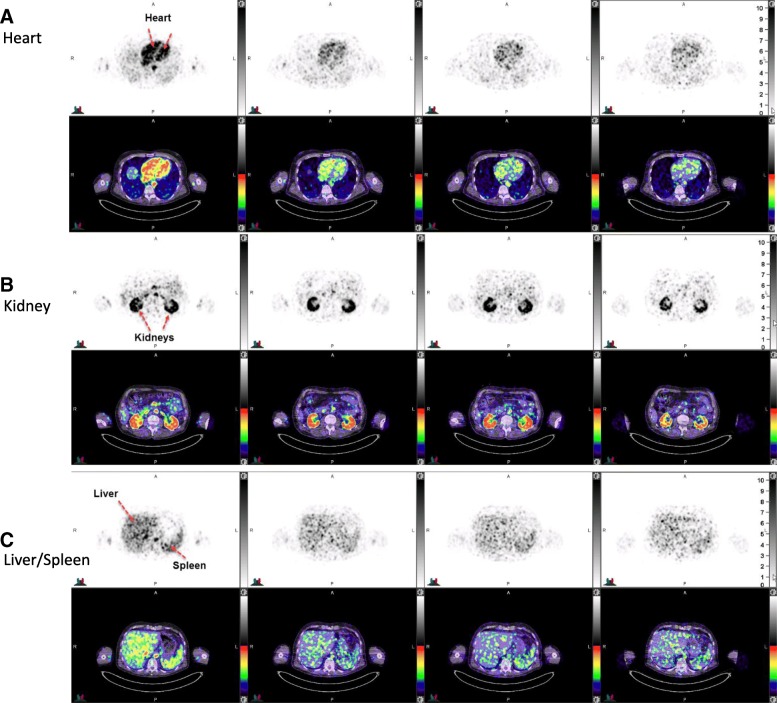
Transaxial images (top PET, bottom PET/CT fused images) across the **a** heart, **b** kidneys, and **c** liver/spleen from the same subject 135 presented in Fig. 1. Times post dose 7, 79, 120, and 168 h post dose. Additional transaxial images for additional subjects 105 and 127 are provided in the Additional file 1: Figures S3 and S5

As expected, the PET/CT images demonstrated an initial dominant vascular distribution, highlighted by signals in cardiac blood pools and large vessels. This was particularly pronounced in images acquired shortly after dosing (Fig. 1). A gradual increased contrast between the kidneys and the rest of the body developed over time. Transaxial images demonstrated a punctate signal, which may reflect noise arising from the low dose of label or local vasculature, as in the study ^89^Zr-GSK3128349 concentration in the blood always exceeded the tissue concentrations, except for prominent uptake in the renal cortex. Features of major organs were still identifiable, as shown for the heart, kidneys, liver, and spleen (Fig. 2). Particularly, the heart chambers and aorta were clearly visible.

### Standardized uptake values

**Fig. 3:**
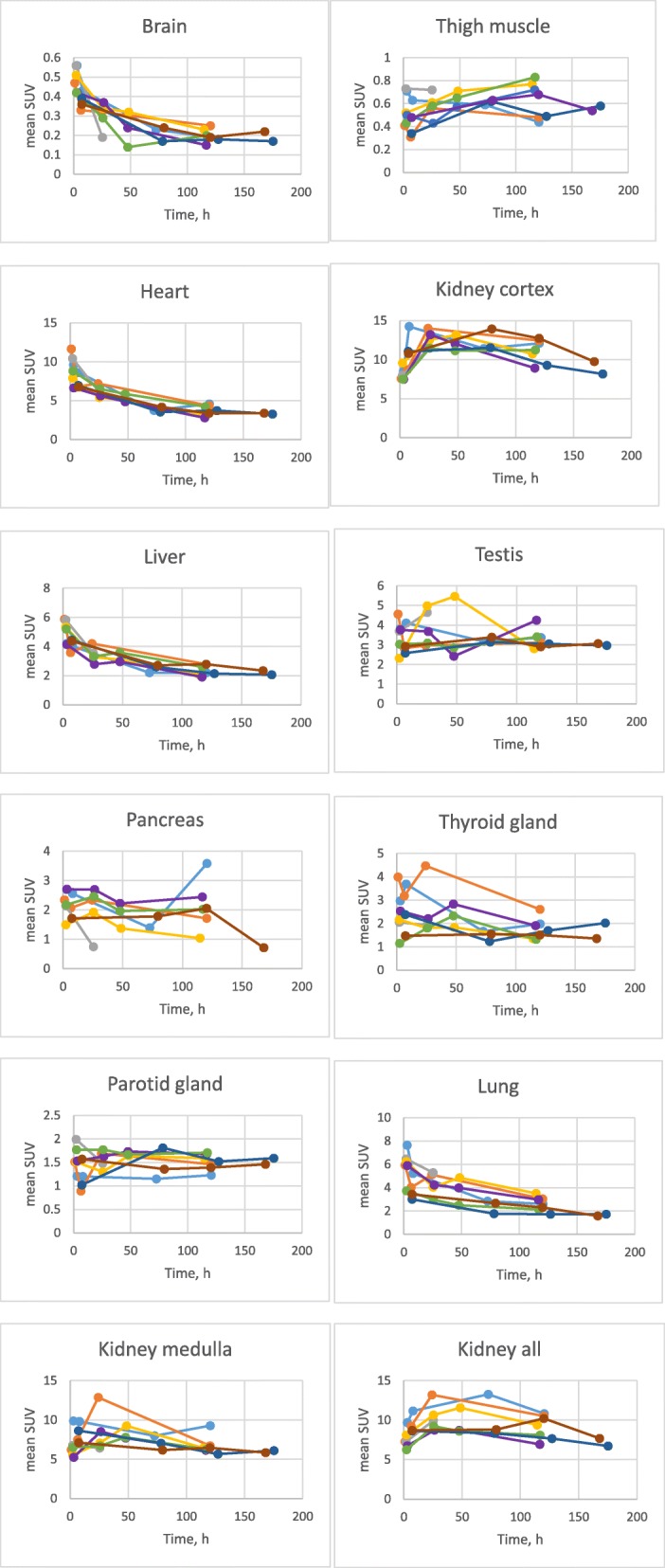
The SUV time course data for [^89^Zr]-GSK3128349 tissue distribution. Individual subjects are identified by the color. While some organs could be measured as whole organs (e.g., kidneys, liver, and spleen), some could only be sampled in part (e.g., muscle and lungs). Subject 105-light blue, 110-orange, 122-gray, 123-yellow, 125-purple, 127-green, 132-dark blue, 135-brown

The mean standard uptake values (SUV) measured by PET/CT characterize the concentration of ^89^Zr-GSK3128349 in the region of interest (ROI) relative to the body average. The mean SUVs ranged from very low values in brain and muscle to high values in blood pools and renal cortex (Fig. 3). In most organs, except muscle and renal cortex, there was a decrease with time. Organ time-activity data across all subjects suggest that tissue distribution steady state is achieved within approximately 100 h after dosing.

### Plasma pharmacokinetics

**Fig. 4:**
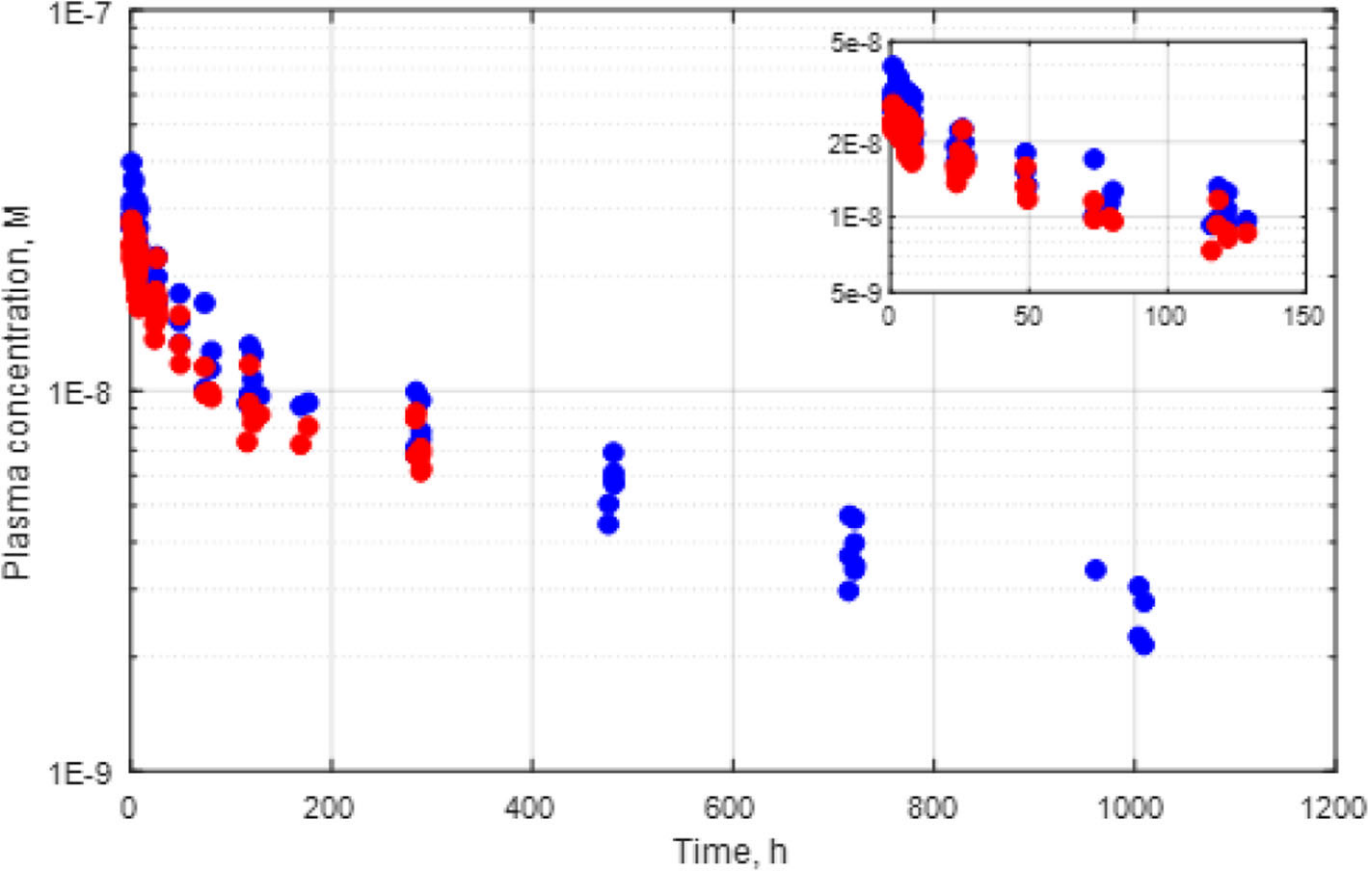
Plasma PK of [^89^Zr]-GSK3128349 as detected by radio PK (red circles), and total GSK3128349 as detected by LC/MS (blue circles). Each dot represents data for a single time point from a single subject

The maximal plasma concentration of GSK3128349 occurred in the first sample drawn at 60 min (390 ± 60 ng/mL), suggesting an initial volume of distribution around 2.5 L, close to that of the plasma volume in humans. The plasma pharmacokinetics of ^89^Zr-GSK3128349 measured by scintillation, and of total GSK3128349 measured by mass spectrometry, followed a biexponential time course (Fig. 4, radio pharmacokinetics (PK) decay-corrected for the ^89^Zr). We found the variation between subjects was small, with only one outlier (at 288 h) excluded from the analysis as it deviated from the mean by 8.4 standard deviations. The initial distribution (alpha) phase, during which the plasma concentration reduced relatively rapidly, lasted for approximately 100 h, and reflects extravasation of the dosed protein into organ interstitial spaces. The beta phase with slower decline was described by first-order kinetics, and characterized as the plasma elimination half-life.

The pharmacokinetic parameters, established through non-compartmental analysis, are presented in Table 2 (and Additional file 1: Table S2). The mean volume of distribution at steady state (Vss) and clearance were 5.4 ± 1.2 L and 9.7 ± 1.6 mL/h, respectively. The terminal half-life of the GSK3128349 AlbudAb was estimated as 422 ± 40 h (17.5 ± 1.7 days); strikingly similar to human albumin [16].

**Table 2:** Plasma pharmacokinetic parameters for total GSK3128349

	AUC (0-inf)	CL	MRT	T½	V_ss
	(ng/mL)*h	mL/h	h	h	mL
Min	81,900	7.8	483	360	4344
Max	128,598	12.2	657	477	6060
Median	97,904	10.2	562	428	5862
Mean	105,482	9.7	565	422	5434
STDEV	17,508	1.6	55	38	696

### Urine excretion of radioactivity

The cumulative urine radioactivity obtained during the 24 h after ^89^Zr-GSK3128349 infusion contained a total of 3.8 and 4.0% of administered radioactivity in the first two subjects. Since 3% in the first two subjects was dictated per protocol as the threshold to initiate further urine sampling, further urine collection (single void samples) was performed in all subsequent subjects. ^89^Zr-GSK3128349 urinary concentration in samples collected beyond 24-h post-dose was about one-third of that in the first 24 h sample (average concentration on day 1 was 495 ± 268 Bq/mL and 178 ± 142 Bq/mL on day 6).

### Tissue-to-plasma ratios

**Fig. 5:**
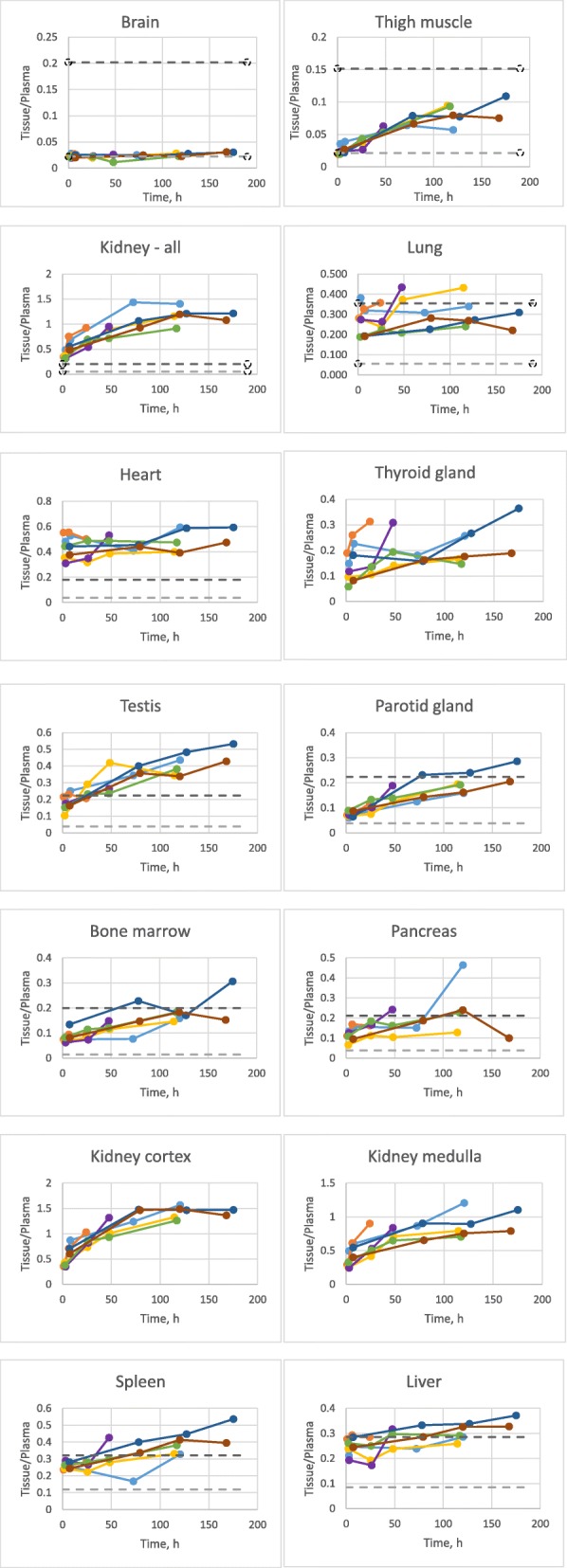
Tissue-to-plasma ratios for different organs across time points for all subjects. The light gray dashed horizontal line denotes the estimated tissue-plasma ratio if the label was confined to the vasculature only. The dark gray horizontal dashed line denotes an estimation for when the label concentration in organ interstitial space is equal to that in plasma. Subject 105-light blue, 110-orange, 122-gray, 123-yellow, 125-purple, 127-green, 132-dark blue, 135-brown

The tissue-to-plasma (t/p) radioactivity concentration ratios for each organ (Fig. 5) predominantly characterize the process of ^89^Zr-GSK3128349 AlbudAb extravasation into interstitial organ spaces. The ratio can change in time, reflective of the rate of the process. We plotted the measured t/p levels from the current study along with the minimal (if the label was confined to the vasculature only) and maximal (if label concentration in organ interstitial space was equal to that in plasma) expected ratios, based on work by Shaw and Betts [14].

We found that in the case of lungs, liver, and spleen, the highest expected t/p ratio was reached quickly, and remained high for the duration of the study, suggesting a rapid distribution of AlbudAb into interstitial spaces in lung, liver, and spleen. In contrast, in brain, the tissue-plasma ratio remained at the theoretical minimum level throughout the study, suggesting that the AlbudAb remained predominantly confined to the brain vasculature.

For numerous organs, the tissue-plasma ratios increased with time, reflecting time-dependent extravasation. Organs in this group were thigh muscle, parotid gland, pancreas, and bone marrow.

Finally, in a few cases, the observed t/p ratio exceeded the theoretical maximum. In heart measurements, the observed value of around 0.5 was significantly higher than the maximal predicted value, being instead closer to that expected for blood. This is expected given that the ROI encapsulated the entire organ including blood present in the heart chambers. In the case of kidneys, the initial value is close to the expected maximum but continues to increase, demonstrating predominance in the cortex. Finally, we also observed significant radiolabel accumulation beyond the theoretical maximum in testis.

## Discussion

The small size of antibody fragments delivers the benefit of improved tissue penetration [17, 18], but also renders them sensitive to rapid renal elimination [19]. Members of the AlbudAb platform, such as GSK3128349, have been engineered to bind albumin with high affinity in several species including humans, and can be genetically fused or chemically conjugated to therapeutic agents (examples include a domain antibody against tumor necrosis factor receptor 1 [20] and apelin [21] as well as the exendin-4 AlbudAb which demonstrated an ability to reduce glucose and insulin in humans [22]). Given that many therapeutic targets are expressed as cell membrane receptors on tissue-embedded cells not exposed directly to plasma, and that GSK3128349 binds albumin also present in the interstitial fluid and lymph, AlbudAbs likely extravasate into organs of the body. To this end, the current PET/CT study of ^89^Zr-GSK3128349 in humans has provided a unique insight into the distribution properties of the AlbudAb platform into various organs. Due to the size of the AlbudAb-albumin complex, the extravasation is likely to be dominated by filtration, like in the case of antibodies [23]. The rate of the process varies from rapid in organs with leaky vasculature (liver, spleen) to slow or imperceptible in the brain. It is likely that the properties of the AlbudAb and albumin will outweigh those of any future potential therapeutic payload, i.e., the drug will remain extracellular and will not diffuse across the plasma membrane. The PK of an AlbudAb conjugated with a therapeutic may well be affected by target-mediated disposition if membrane proteins are targeted by the therapeutic agent. More quantitative descriptions of tissue penetration and target engagement will be possible for specific therapeutic AlbudAbs within the framework of physiologically based pharmacokinetics [14, 19, 23].

This study included four adaptively modified PET/CT scan time-points per subject that yielded tissue penetration and elimination PK using only eight subjects. Plasma concentration time course data obtained via LC-MS/MS compared to radiolabel PK yielded very similar results, suggesting that the presence of the radiolabel (on only ~ 0.015% of the molecules dosed) does not affect the tissue distribution properties of GSK3128349. The initial volume of distribution (calculated from the plasma Cmax) was very close to human plasma volume, indicating that the IV dose is rapidly diluted in the circulation after dosing. A process significantly faster than the subsequent tissue distribution phase reaching steady-state in about 100 h, similar to that reported for albumin [16] and other large proteins such as intact monoclonal antibodies [24]. The 5.5 L steady-state volume of distribution for GSK3128349 is also typical for monoclonal antibodies whose distribution is unaffected by target-binding [25, 26]. Finally, the terminal half-life of GSK3128349 of 17.5 days is close to the 18–19 days reported for human albumin [27], implying that AlbudAb remains bound to albumin during endosomal recycling [28] and hence probably shares the albumin elimination pathway. Overall, the AlbudAb plasma kinetics properties described here are very similar to that of albumin, suggesting that the current AlbudAb PET study data may provide an indirect assessment of albumin distribution.

T/p ratios for several organs are significantly higher than expected from their respective organ vascular spaces alone but are instead more consistent with a summed combination of vascular and interstitial spaces. Although t/p ratios did not always reach a level to suggest a similar concentration in the vascular and interstitial compartments, this observation is consistent with Wiig et al. and Gyenge et al. [29–31] who have shown that there is an “exclusion space” in tissue on the order of 16–26% for albumin and antibodies, whereby a portion of the interstitial space is not accessible. “Exclusion space” estimates have only been made for a few organs, therefore our comparisons have used the estimated maximum t/p ratios (Fig. 5) based on an assumption that GSK3128349 can distribute into the full interstitial space with a concentration equal to that in plasma. However, filtration at the vascular barrier is expected to reduce the interstitial concentrations of plasma proteins in organs with continuous and fenestrated capillaries [32]. In addition, there can be a reduction in interstitial tissue volume for larger proteins, and therefore, the actual highest attainable t/p ratios could be lower than that presented herein. Consistent with this, the steady-state volume of distribution measured in this study for GSK3128349 is twofold higher than the plasma volume, whereas the entire extravascular volume exceeds that of plasma by about fourfold, suggesting that GSK3128349 achieves around a two- to fourfold dilution during the extravasation process.

The data reported here suggest that GSK3128349 has a rapid access to the tissue interstitium in some organs (e.g., lung and liver due to fenestrated endothelium) and is virtually excluded from others (e.g., brain by the properties of the blood-brain barrier). The t/p ratio time course for ^89^Zr-GSK3128349 increases with time in several organs—especially noticeable in muscle, renal cortex, bone marrow, parotid gland, testis, and spleen which is likely to reflect the penetration of GSK3128349 into the interstitial space in these organs. The data are not sufficient to elucidate the exact mechanism for transfer from plasma to interstitium, but GSK3128349 bound to albumin can be expected to be transported across the capillary endothelium by filtration and diffusion, as described within the framework of two-pore hypotheses of extravasation [32].

PET measures the total tissue concentration of ^89^Zr, comprised of both the intact ^89^Zr-GSK3128349 protein in both vascular and interstitial spaces, as well as any potential ^89^Zr-containing degradation products due to the residualizing nature of the label. This is likely to be especially relevant in the case of kidneys, where the cellular uptake and catabolism of ^89^Zr-GSK3128349, as well as specific uptake and intracellularly retention of any residual free ^89^Zr, can lead to accumulation of the label [33, 34] and contribute to the overall signal.

We observed high PET signal in kidneys with an average final SUV of approximately 9, and even higher levels in the cortical region. The kidneys constitute less than 1% of body weight, indicating that the fraction of administered radioactivity captured by the kidneys is about 9%. At the same time, we found 3–4% of the administered radioactivity in the initial 24-h urine, which is the typical daily range for albumin passing through the glomerular barrier in humans [35]. Most protein found in urine is thought to be endocytosed via megalin-cubilin-mediated uptake in the proximal tubules of the renal cortex and to be degraded [35]. Our imaging data, which indicate significant accumulation of ^89^Zr in the renal cortex are in agreement with this given the residualizing nature of ^89^Zr label. Importantly in support of this residualization hypothesis, no such renal accumulation is observed in pre-clinical species, when the AlbudAb is conjugated to a non-residualizing radioisotope, e.g., ^3^H ([17] & manuscript in preparation). Nevertheless, we cannot eliminate the possibility that the dosed sample contained a small amount of free label which may have been rapidly removed in the kidneys and in part retained and passed on to urine soon after dosing.

Our attachment of ^89^Zr to the GSK3128349 AlbudAb can be considered as representative of a therapeutic payload that could be replaced instead by other protein domains through genetic fusion, or by coupling of peptides or even small molecule drugs. One advantage of AlbudAbs compared with full sized antibodies is that around a tenfold higher molar concentration can be achieved following the same mg/kg dose due to their relatively low molecular weights. This advantage can be critically important, particularly if a target of interest resides in a difficult to reach tissue compartment.

Extending the plasma half-life of small molecule drugs by conjugation of an AlbudAb also offers the option of maintaining required plasma exposures at much lower doses of active compound than otherwise possible; potentially reducing the risk of systemic toxicity driven by higher peak plasma concentrations, more frequent dosing and increased total dose administered.

The nature, location, concentration, and the turnover of the therapeutic target can also be expected to affect both the kinetics and tissue concentrations of AlbudAb-based therapeutics. While no major deviations from the described AlbudAb PK and tissue distribution would be expected in the case of soluble targets, in the case of binding to cell surface proteins, one would expect to see target-mediated disposition, just like for conventional monoclonal antibodies. An understanding of the distribution of the unconjugated AlbudAb, as provided here through PET imaging, can now be incorporated with the understanding of target-mediated disposition activities of specific targets of interest to model target engagement of candidate therapies in support of both discovery and development efforts.

## Conclusions

The study has confirmed the utility of ^89^Zr-immuno-PET as a versatile method to explore tissue distribution and organ access for proteins, demonstrating that it is possible to conduct an immuno-PET study in healthy volunteers at radiation exposure below 10 mSv. The average administered radioactivity of 14 MBq was very low but generated adequate images and reasonable quantitative information up to 7 days after administration allowing measurements of tissue penetration and elimination PK of ^89^Zr-GSK3128349.

The free fraction of GSK3128349 AlbudAb is expected to be in the region of ≈ 0.002% in plasma and in these conditions GSK3128349 acquires albumin-like properties in circulation and in organ extravascular compartments. These data contrast with shorter residence times observed for other similarly sized dAbs and smaller proteins that do not bind albumin and can be eliminated within hours by renal filtration.

The study also demonstrated significant differences between organs in respect of the kinetics of GSK3128349 AlbudAb extravasation, indicating that steady-state exposure in different organs may be different from that achievable with single doses. The results suggest rapid access to tissue interstitium in the liver, lungs, spleen, bone marrow, and other organs, with no extravasation and accumulation in the brain. Hence, the albumin-binding and distribution properties suggest that GSK3128349 has the potential to be a useful platform to carry therapeutic payloads to access tissues (with exception of CNS) with desirable pharmacokinetics.

## Additional file

Additional file 1: **Materials.** AlbudAb production and radiolabeling. **Materials.** Quantification of plasma GSK3128349 by LC-MS/MS. **Methods.** PET image analysis. **Methods.** Subject cohort inclusion and exclusion criteria. Figure S1. Study outline and procedure. **Table S1.** Subject details. **Table S2.** Pharmacokinetic parameters. **Figure S2.** Maximum intensity projection, subject 127. **Figure S3.** PET/CT images, subject 127. **Figure S4.** Maximum intensity projection, subject 105. **Figure S5.** PET/CT images, subject 105. (DOCX 3305 kb)

## Abbreviations

^89^Zr: Zirconium-89
AlbudAb: Albumin-binding domain antibodies
BMI: Body mass index
cGMP: Current Good Manufacturing Practices
dAb: Domain antibodies
ECL: Electrochemiluminescent
IV: Intravenous
LC-MS/MS: Liquid chromatography tandem mass spectrometry
MBq: Mega becquerel
MIP: Maximum intensity projections
mSv: Millisievert
NCA: Non-compartmental analysis
PET: Positron emission tomography
PET/CT: Positron emission tomography/computed tomography
PK: Pharmacokinetics
PSF: Point-spread function
ROI: Region of interest
SUV: Standard uptake values
t/p: Tissue-to-plasma
TOF: Time-of-flight
VoI: Volumes of interest
Vss: Volume of distribution at steady state
