# Supplementary material for: The biodistribution and clearance of AlbudAb, a novel biopharmaceutical medicine platform, assessed via PET imaging in humans
Source: EJNMMI Res. 2019 May 21;9:45. doi: 10.1186/s13550-019-0514-9 (PMC6529487; doi:10.1186/s13550-019-0514-9)
Supplement: Supplementary file 1 — Materials. AlbudAb production and radiolabeling. Materials. Quantification of plasma GSK3128349 by LC-MS/MS. Methods. PET image analysis. Methods. Subject cohort inclusion and exclusion criteria. Figure S1. Study outline and procedure. Table S1. Subject details. Table S2. Pharmacokinetic parameters. Figure S2. Maximum intensity projection, subject 127. Figure S3. PET/CT images, subject 127. Figure S4. Maximum intensity projection, subject 105. Figure S5. PET/CT images, subject 105. (DOCX 3305 kb) [file 13550_2019_514_MOESM1_ESM.docx]

# The biodistribution and clearance of AlbudAb, a novel biopharmaceutical medicine platform, assessed via PET imaging in humans

Kevin S. Thorneloe^1^, Armin Sepp^1^, Sean Zhang^1^, Laura Galinanes-Garcia^1^, Paul Galette^1^, Wasfi Al-Azzam^1^, Danielle J. Vugts^2^, Guus van Dongen^2^, Phillip Elsinga^3^, Johan Wiegers^3^, Andor W.J.M. Glaudemans^3^, Veena Vincent^1^, Jessica Renaux^1^, Matt Szapacs^1^, Mary Birchler^1^, Matthew Cleveland^1^, Mats Bergstrom^1^, Marie Davies^1^

Affiliations: ^1^ GSK, ^2^VU University Medical Center, ^3^University of Groningen, University Medical Center Groningen

Contact details: Marie Davies, GSK, Gunnels Wood Road, Stevenage, UK SG1 2NY. Telephone +44 7887 055415. E-mail: [marie.l.davies@gsk.com](mailto:marie.l.davies@gsk.com).

# Supplementary data

## AlbudAb production and radiolabeling

^89^Zr-GSK3128349 was produced in compliance with current Good Manufacturing Practice at the VU University Medical Center. The procedure of radiolabelling and quality control of GSK3128349 with ^89^Zr have been validated. ^89^Zr-GSK3128349 has been produced according to previously reported method of Verel et al. [1]. In short, GSK3128349 (110 µL of 5 mg/mL (50 nmol) was diluted to 0.55 mg/mL with 0.9% NaCl (870 µL), after which the pH is adjusted to 9.5-9.7. 2 equivalents of Fe-TFP-N-suc-desferal in acetonitrile (20 µL of 5 nmol/µL, 100 nmol) were added, mixed carefully and reacted for 30 minutes at room temperature. Next, 50 µL of 100 mg/mL gentisic acid pH 4.0-4.2 was added, followed by adjustment of the pH to 4.20-4.50 with 0.25 M sulfuric acid. Hereafter, EDTA (50 µL, 25 mg/mL) was added and reacted for 30 minutes at 35˚C, after which the conjugated DFO-GSK3128349 was purified by size exclusion chromatography (PD10, GE Healthcare) and the product collected in 50 mM NaOAc + 200 mM sucrose pH 5.50±0.30.

Finally, *N*-suc-desferal-GSK3128349 was radiolabelled. To this end 100 µL 1M oxalic acid containing the required amount of ^89^Zr was mixed with 45 µL 2M Na_2_CO_3_ and reacted for 3 minutes. Next 0.5 mL 0.5 M Hepes and 0.355 mL *N*-suc-desferal-GSK3128349 were added and reacted for 60 minutes at room temperature while slowly shaken. After the incubation period ^89^Zr-GSK3128349 was purified by size exclusion chromatography using a PD10 column. The product was eluted in 50 mM NaOAc + 200 mM sucrose pH 5.50 ± 0.30. The product was formulated to arrive at an injection dose of 15 MBq – 1 mg – 10 mL ^89^Zr-GSK3128349. An average of 30% was included in the formulation to deliver the required dose. The mean of the product pH was 5.85 ± 0.02. The mean radiochemical purity as assessed by iTLC was 97.7 ± 0.6%. To this end 2 µL of product was applied on a TLC strip (Biodex, cat nr. 150-771) and developed in 10% acetonitrile in 20 mM citric acid+ 50 mM EDTA pH 4.8-5.0 as described by the supplier. ^89^Zr-GSK3128349 remained on the baseline, while impurities such as free ^89^Zr and ^89^Zr-DFO run with the solvent front. The mean protein integrity was 100% as determined by size exclusion HPLC using a BioSEC3 150 Å, 7.8x 300 mm, 3 µm, (Agilent) using a mixture of 150 mM sodium phosphate buffer and 10% isopropanol in water as the eluent at a flow rate of 0.5 mL/min. The mean immune reactive fraction as assessed by a binding assay was 84.1 ± 3.6 %. Sterility of each ^89^Zr-GSK3128349 batch was assured by performing a media fill immediately after final filter sterilisation of each batch. These procedures resulted in a sterile final product with endotoxin levels <0.4 EU/mL.

The structural integrity of ^89^Zr-GSK3128349 was monitored by SE-HPLC at 280 nm to confirm the presence of ≥ 90.0 % intact monomer. The albumin binding characteristics of ^89^Zr-Zr-GSK3128349 were confirmed using human serum albumin (HSA) - coated beads (Sovicell, Leipzic, Germany) at five concentrations ranging from 512 nM to 2 nM per tube. ^89^Zr-GSK3128349 was added at 30 ng/mL and incubated overnight at 2-8°C, followed by excess unlabelled GSK3128349 to determine non-specific binding. The next day, the beads are spun down and half of the supernatant is transferred to a new tube. The immunoreactive fraction of ^89^Zr-GSK3128349 that was bound to the beads was always excess of ≥ 70.0% for each sample preparation.

**Quantification of plasma GSK3128349 by LC-MS**

The LC-MS/MS method for the quantitation of the non-conjugated AlbudAb (GSK3128349) from 50 µL of human plasma is based on enzymatic digestion, using trypsin, followed by solid phase extraction and quantitation of the resulting representative peptide ‘LLILAFSR’ by LC-MS/MS using an electrospray interface and multiple reaction monitoring. The tryptically derived representative peptide ‘LLILAFSR’ was selected from the complimentary determining region of the AlbudAb molecule. For quantitation, a calibration curve was constructed by spiking the non-conjugated Albudab over the range of 25 to 5000 ng/mL into blank human plasma. This was followed by spiking of a isotopically labeled version of the non-conjugated Albudab into plasma. Digestion of the plasma samples was performed using 4 µg of trypsin overnight at 37 °C in 200 µL 80:20 50 mM ammonium biocarbonate:acetonitrile. Solid phase extraction of the tryptic peptides was peformed using strong cation exchange (Strata XC 30 mg/well from Phenomenex) followed by liquid chromatography of the purified peptides using a Waters UPLC equipped with a Waters HSS T3 1.8 µm, 2.1 x 50 mm column with an initial mobile phase composition of 90% formic acid (0.1%) in water and 10% acetonitrile that was increased to 25% over the first 3 minutes of the method (typical retention time is 2.6 min). The mass transitions monitored were 466 m/z  480 m/z corresponding to the [M + 2H]2+ to y4 ions for the ‘LLILAFSR’ peptide using a Waters Xevo TQ-S mass spectrometer. During validation the method was shown to be precise (≤20.6%), accurate (≤17.1%) and selective for the peptide ‘LLILAFSR’ in human plasma with a range of 25 to 5000 ng/mL.

**PET image analysis**

Analysis of the PET images was performed using Siemens Syngo software for region of interest delineation, which allows for semiautomatic contours and quantification in these regions (i.e. SUVmax, SUVmean, SUVpeak).  ROIs were drawn either directly onto the PET images, or were delineated on low dose CT and transferred to the PET images to extract results. In a preprocessing step the low dose CT data were resampled to the PET voxel size and PET FOV.  Where technically feasible, the following organs were included in the analysis:

| Organ | Contour |
| --- | --- |
| Liver | Large organ ROI approximating total organ |
| Spleen | Large organ ROI approximating total organ |
| Muscle (Left/Right Thigh) | Large ROI |
| Brain | Large organ ROI approximating total organ |
| Lung (Left/Right) | Large organ ROI approximating total organ |
| Parotids | Organ ROI approximating total organ |
| Thyroid | Organ ROI approximating total organ |
| Whole Heart | Large organ ROI approximating total organ |
| Aorta (whole blood) | Fixed size VOI placed in the middle of the aortic arch (axial view) on five consecutive images |
| Thymus | If present, organ ROI approximating total organ |
| Bone Marrow | Fixed size ROI placed on 3 consecutive vertebrae |
| Pancreas | Organ ROI approximating total organ |
| Renal cortex (Left/Right) | Organ ROI approximating total organ |
| Renal Medulla (Left/Right) | Organ ROI approximating total organ |
| Kidneys (Left/Right) | Large organ ROI approximating total organ |
| Bladder | Large organ ROI approximating total organ |
| Testis | Organ ROI approximating total organ |
| Whole body | Entire area of scanning coverage |

**Subject cohort inclusion and exclusion criteria**

Inclusion criteria: A subject was eligible for inclusion in this study only if all of the following criteria applied:

- Healthy males between 50 and 65 years of age inclusive, body mass index (BMI) within the range 19.0 to 31.0 kg/m2 (inclusive).
- A subject with a clinical abnormality or laboratory parameters outside the reference range for the population being studied could be included only if the Investigator and the GSK Medical Monitor agreed that the finding was unlikely to introduce additional risk factors and did not interfere with the study procedures.
- Aspartate aminotransferase (AST), alanine aminotransferase (ALT), alkaline phosphatase and bilirubin ≤1.5x Upper Limit Normal (ULN) (isolated bilirubin >1.5xULN is acceptable if bilirubin is fractionated and direct bilirubin <35%).
- Average Corrected QT interval (QTc) ≤450 msec. Subjects agreed to use contraception from the time of the first dose of study treatment until 100 days post dose.

Exclusion criteria:

- Current evidence or history of influenza-like illness as defined by fever (>38^o^C) and two or more of the following symptoms within 7 days before dosing: cough, sore throat, rhinorrhea, sneezing, limb/joint pain, headache, vomiting/diarrhea, liver disease, or known hepatic or biliary abnormalities except Gilbert's syndrome or asymptomatic gallstones, acute renal failure, known renal disease, or a renal disorder or abnormality that might compromise renal function. This included having one kidney.
- Previous inclusion in a research and/or medical protocol involving nuclear medicine, PET or radiological investigations or occupational exposure that, together with the proposed study, and could result in a total radiation exposure greater than 10 mSv over a 3 year period.
- Habits: History of regular alcohol consumption within 6 months of the study defined as an average weekly intake of >21 units. Smoker with ≥5 cigarettes/day or with a smoking history of >5 pack years
- Unable to refrain from the use of prescription drugs within 7 days or 5 half-lives (whichever was longer) prior to the first dose of study treatment, unless in the opinion of the Investigator and GSK Medical Monitor the medication did not interfere with the study procedures or compromise subject safety.
- History of sensitivity to any of the study treatment or components or a history of drug or other allergy that, in the opinion of the investigator or GSK Medical Monitor, contraindicated their participation.
- Claustrophobia that would limit the ability to remain still in the PET/CT scanner for the required amount of time to complete the scanning protocol.
- Subject had metal present in their body that would interfere with the PET/CT scanning.
- Estimated glomerular filtration rate (eGFR) <60 mL/min/1.73 m2 (utilizing the Chronic Kidney Disease Epidemiology Collaboration (CKI-EPI) equation).
- Urine mg protein/mg creatinine Urine Protein Creatinine Ratio (UPCR) >0.3.
- Evidence of haematuria by urinalysis (1+ or greater dipstick test).
- Positive pre-study Hepatitis B surface antigen or positive Hepatitis C (Hep C) antibody result within 3 months of screening.
- A positive test for Human Immunodeficiency Virus (HIV) antibody.
- A positive pre-study drug/alcohol screen.
- The subject had participated in a clinical trial and had received an investigational product within the following time period prior to the first dosing day in the current study: 30 days, 5 half-lives or twice the duration of the biological effect of the investigational product (whichever is longer).
- Exposure to more than four new chemical entities within 12 months prior to the first dosing day.
- Where participation in the study could result in donation of blood or blood products in excess of 500 mL within a 56 day period.


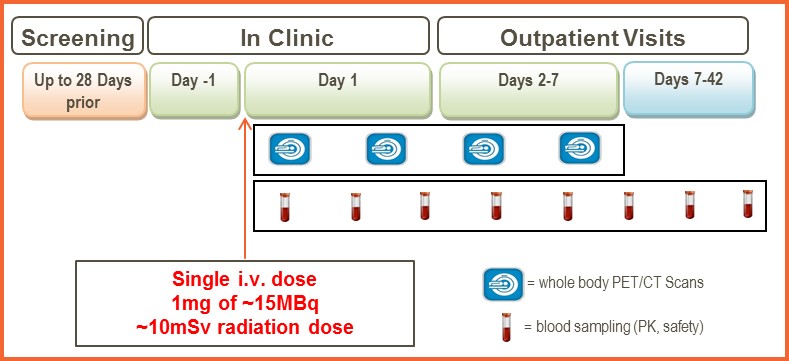


**Figure S1**. General outline of the study and its procedures

**Table S1.** Eight Caucasian male subjects were enrolled into the study. All subjects had four PET/CT scans, except subject 122 who declined further participation after the first two scanning sessions for availability reasons

| Subject | Age (years) | Weight (kg) | BMI^*^ (kg/m^2^) | Dose of radioactivity (MBq) | Colour in Main Figures 3 and 5 |
| --- | --- | --- | --- | --- | --- |
| 105 | 54 | 76.1 | 25.7 | 12.77 | Light blue |
| 110 | 53 | 72.2 | 22.8 | 14.53 | Orange |
| 122 | 54 | 68.6 | 23.5 | 14.24 | Grey |
| 123 | 60 | 92.3 | 27.6 | 13.27 | Yellow |
| 125 | 53 | 91.8 | 27.4 | 14.64 | Purple |
| 127 | 56 | 70.2 | 21.4 | 14.72 | Green |
| 132 | 54 | 73.0 | 20.9 | 15.03 | Dark blue |
| 135 | 56 | 83.1 | 23.3 | 13.06 | Brown |

^*^Body mass index

**Table S2.** Plasma pharmacokinetic parameters for ^89^Zr-GSK3128349 calculated from the data for seven subjects out of eight who completed the study (n=7). AUC-Area Under the Curve, STDEV-Standard deviation, CL-clearance, MRT-mean residence time, t_½_-half-life, V_ss- steady state volume of distribution

|  | ^89^Zr-GSK3128349 | | | | |
| --- | --- | --- | --- | --- | --- |
|  | AUC(0-inf) | CL | MRT | t_½_ | V_ss |
|  | (Bq/mL)*h | mL/h | h | h | mL |
| Min | 782949 | 9.4 | 261 | 178 | 4183 |
| Max | 1322742 | 15.0 | 773 | 555 | 7308 |
| Median | 877736 | 14.2 | 381 | 265 | 5172 |
| Mean | 945975 | 13.6 | 429 | 300 | 5550 |
| STDEV | 197158 | 2.4 | 170 | 130 | 1197 |

**Additional subject images**


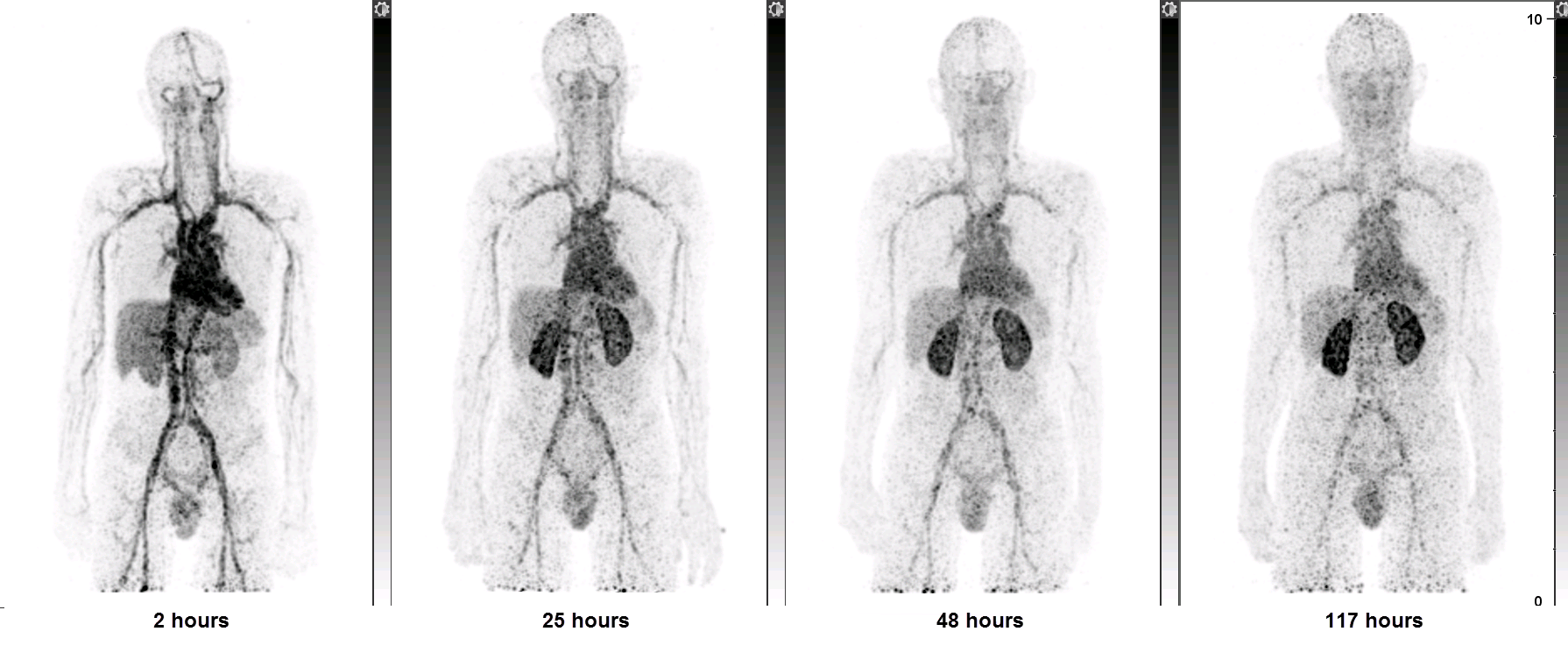


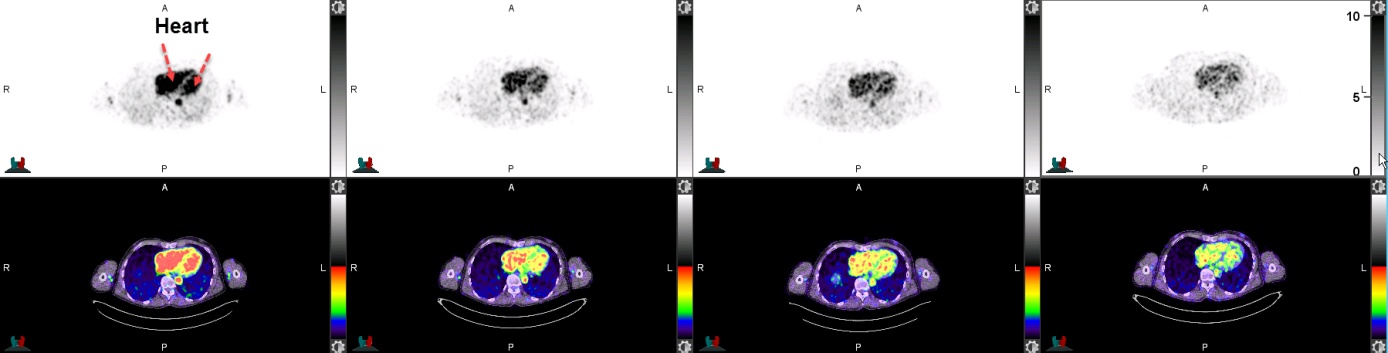
**Figure S2:** Maximum intensity projection (MIP) images across visits for subject 127


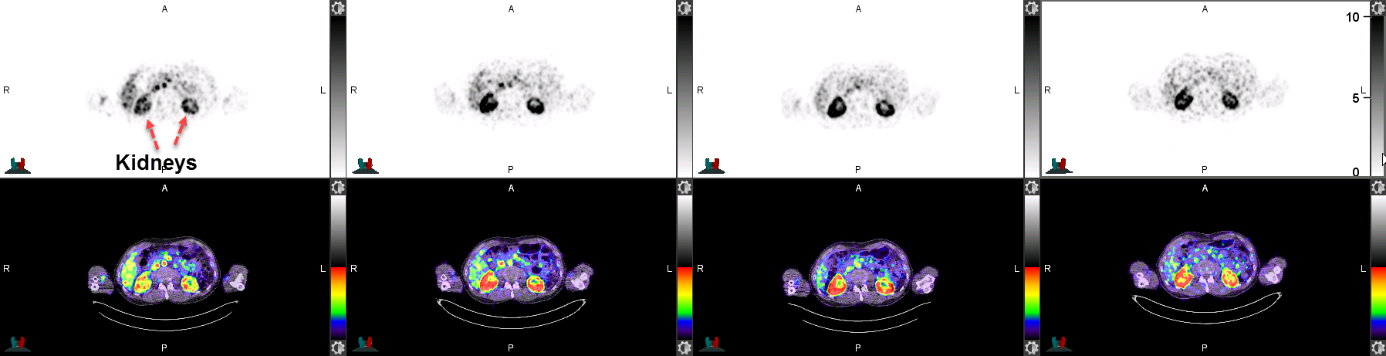

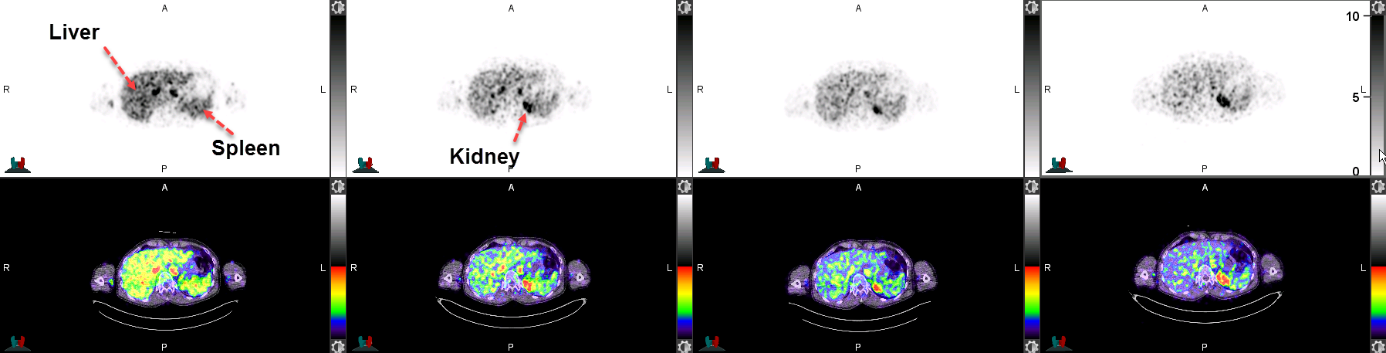

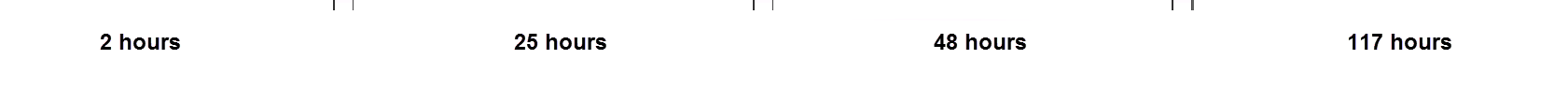


A) Heart

B) Kidney

C) Liver/Spleen

**Figure S3:** Axial PET (top) and PET/CT fused images across the (A) heart,(B) kidneys and (C) liver/spleen from subject 127


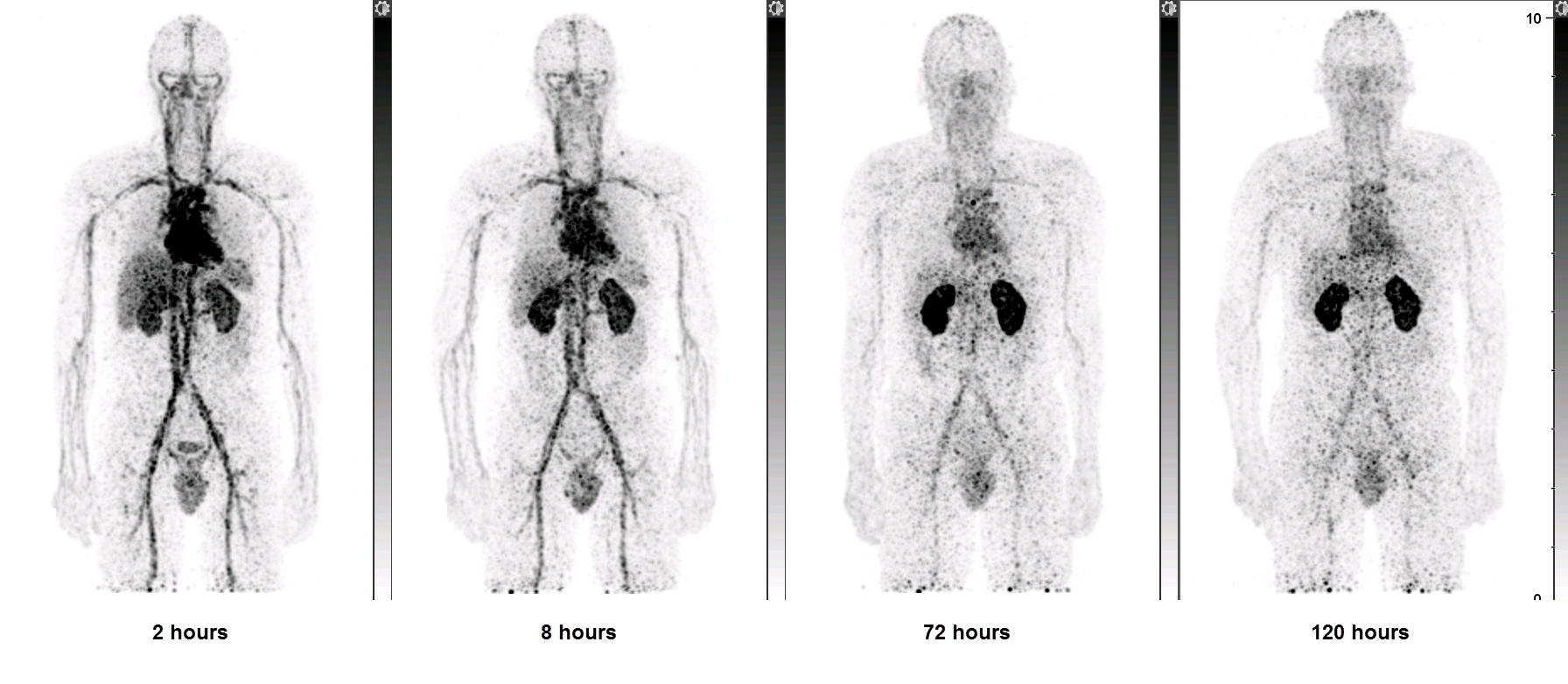


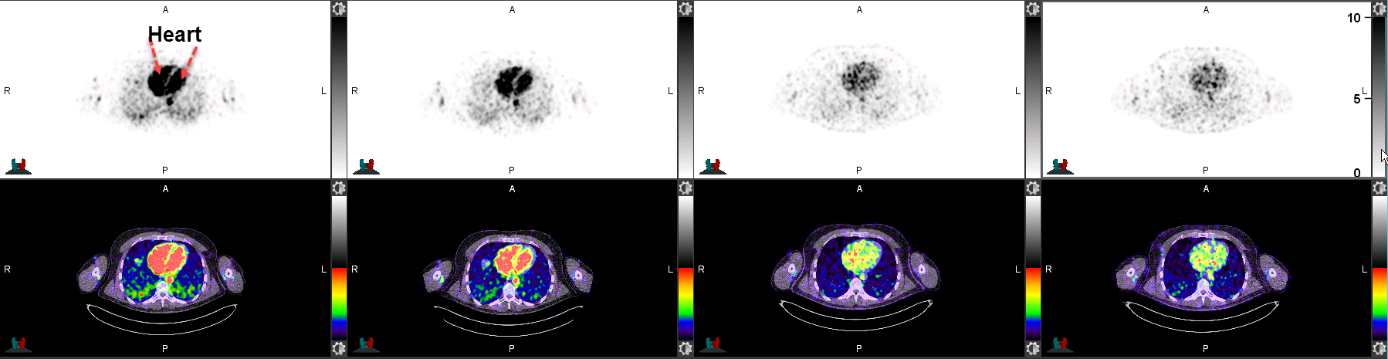
**Figure S4:** Maximum intensity projection (MIP) images across visits for subject 105


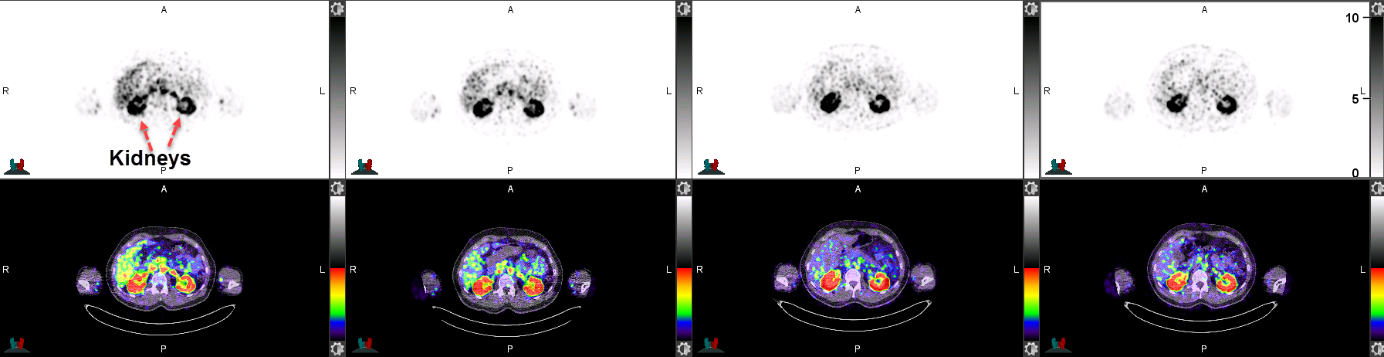

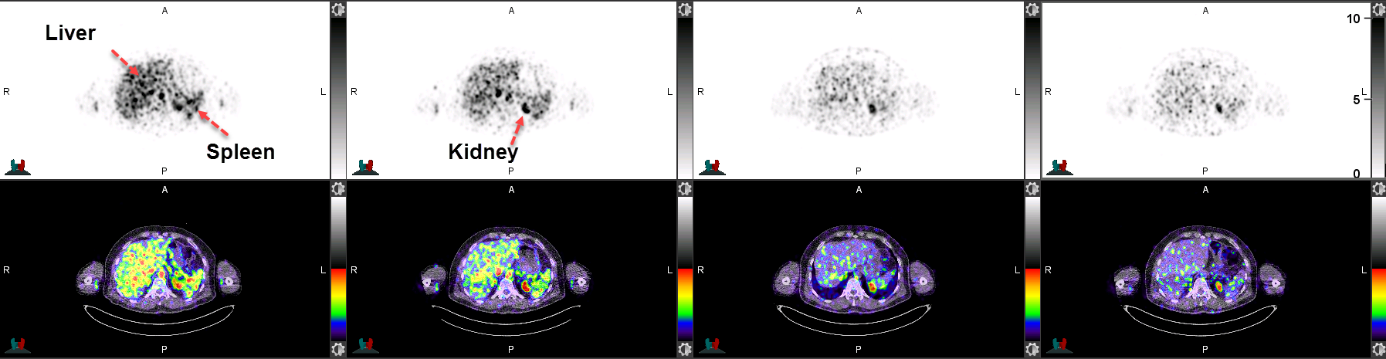

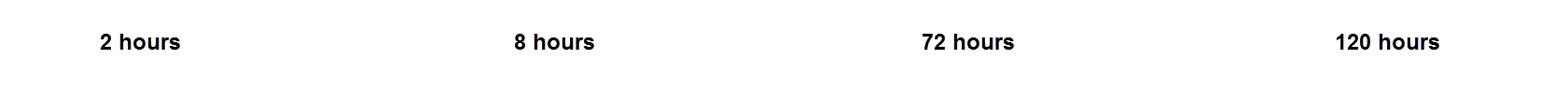


A) Heart

B) Kidney

C) Liver/Spleen

**Figure S5:** Axial PET (top) and PET/CT fused images across the (A) heart,(B) kidneys and (C) liver/spleen from subject 105

**References**

1. Verel I, Visser GWM, Boellaard R, Stigter-van Walsum M, Snow GB, van Dongen GAMS. 89Zr Immuno-PET: Comprehensive Procedures for the Production of 89Zr-Labeled Monoclonal Antibodies. Journal of Nuclear Medicine. 2003;44:1271-81.
